# Associations of the inflammasome-dependent cytokine IL-18 with risk of coronary heart disease

**DOI:** 10.1093/cvr/cvag135

**Published:** 2026-06-23

**Authors:** Harry Björkbacka, Isabel Gonçalves, Gunnar Engström, Alexandru Schiopu, Anna von Sydow, Maria Ryaboshapkina, Vijayalakshmi Varma, Alastair Moss, Angela C Shore, Andrea Natali, Faisel Khan, Jan Nilsson

**Affiliations:** Department of Clinical Sciences Malmö, Lund University, Sweden; Department of Clinical Sciences Malmö, Lund University, Sweden; Department of Cardiology, Skåne University Hospital, Jan Waldenströms gata 35, 21428 Malmö, Sweden; Department of Clinical Sciences Malmö, Lund University, Sweden; Department of Translational Medicine, Lund University, Malmö, Sweden; Nicolae Simionescu Institute of Cellular Biology and Pathology, Bucharest, Romania; Department of Internal Medicine, Skåne University Hospital, Lund, Sweden; AstraZeneca, Mölndal, Sweden; AstraZeneca, Mölndal, Sweden; AstraZeneca, Gaithersburg, USA; AstraZeneca, Cambridge, UK; Diabetes and Vascular Medicine, University of Exeter Medical School and NIHR Exeter Clinical Research Facility, Exeter, UK; Department of Clinical and Experimental Medicine, University of Pisa, Pisa, Italy; Division of Cardiovascular Research, University of Dundee, Dundee, UK; Department of Clinical Sciences Malmö, Lund University, Sweden

**Keywords:** Inflammasome, Myocardial infarction, Atherosclerosis, Biomarkers, Drug targets

## Abstract

**Aims:**

Inflammasome activation has emerged as a promising therapeutic target to reduce residual cardiovascular (CV) risk in patients on guideline lipid-lowering therapy. IL-1β and IL-6 have been intensively studied, whereas the role of the inflammasome-related cytokine IL-18 has gained less attention. Here we aimed to assess the associations of IL-18 with risk of coronary events and severity of atherosclerosis.

**Methods and results:**

The study included 4742 participants participating in a prospective Swedish Malmö Diet and Cancer (MDC) cohort study, 54 219 general population participants in the UK Biobank, and 1500 participants with Type 2 diabetes and/or known CV disease in the European multicentre SUMMIT VIP imaging study. Plasma levels of IL-18 were analysed using proximity extension assay and atherosclerosis by carotid ultrasound. The incidence of MI and CV death was registered during a 23-year follow-up for the MDC cohort and for a 15-year follow-up for the UK Biobank cohort. There were 617 cases of MI and 644 CV deaths in the MDC cohort and 2454 cases of MI and 1537 CV deaths in the UK Biobank cohort. Elevated IL-18 was associated with an increased risk of MI and CV death independent of hsCRP and IL-6 in both cohorts. In Cox proportional hazards regression models, this association was independent of major CV risk factors in the UK Biobank but not in the MDC cohort. IL-18 did not provide clinically meaningful risk reclassification in the UK Biobank cohort. The association of IL-18 with carotid atherosclerosis in the SUMMIT VIP cohort was weaker than that of IL-6.

**Conclusion:**

IL-18 is independently associated with risk of MI and CV death in the general population but does not improve clinical risk stratification. Targeting IL-18 directly or through inflammasome inhibition represents a promising approach for treatment of residual inflammatory risk in high-risk atherosclerosis patients that merits to be evaluated in future randomized clinical trials.

## Graphical Abstract

**Graphical Abstract:**
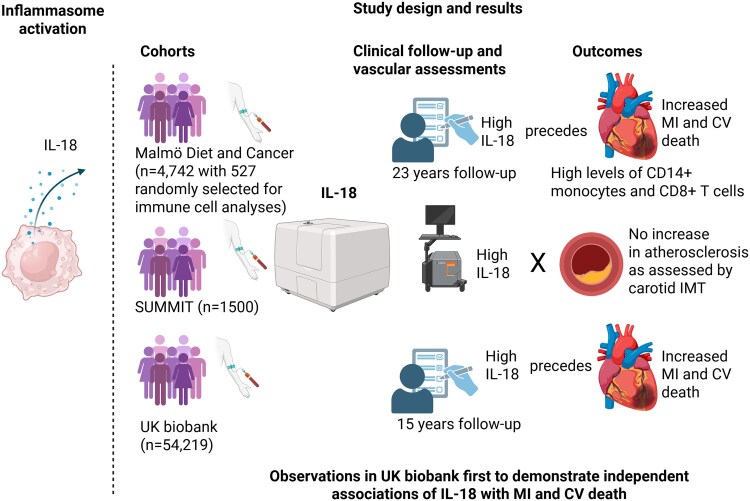

**Time of primary review: 20 days**

## 1. Introduction

A recent collaborative analysis of three large cardiovascular (CV) intervention trials involving 31 245 statin-treated patients showed that those in the highest CRP quartile had a markedly higher risk of MI and CV death than those in the highest quartile of low-density lipoproteins (LDL) cholesterol indicating that inflammation was the main residual risk factor in these patients.^1^ Several large randomized clinical trials including CANTOS, COLCOT, and LoDoCo2 have shown that anti-inflammatory therapies can reduce CV risk in patients on guideline lipid-lowering medication.^2–4^ The CANTOS participants and a representative subset of COLCOT participants had elevated hsCRP >2 mg/dL at baseline, whereas CRP levels were not assessed in LoDoCo2. The therapies evaluated in these trials, canakinumab and colchicine, target factors associated with inflammasome activation. The inflammasomes are a family of multiprotein complexes that, when activated by pathogens and endogenous damage-associated molecular patterns, trigger the activation and release of the pro-inflammatory cytokines IL-1β and IL-18.^5^ IL-1β is a potent inducer of IL-6 which in turn stimulates production of the acute phase reactant C-reactive protein (CRP) in the liver.^6^ Both IL-6 and CRP [measured by high-sensitivity (hs) tests] are well-established markers of CV risk, but only IL-6 is considered to be causally associated with cardiovascular disease (CVD).^7,8^ Several CV outcome trials evaluating the effect of IL-1β and IL-6 inhibition have been completed or are ongoing,^9^ whereas the role of IL-18 as a possible drug target has gained less attention. Experimental studies in mouse models of atherosclerosis have shown that IL-18 promotes atherosclerosis,^10,11^ but it remains unclear if these findings are relevant in humans. We asked the question whether IL-18 predicts incident CV events in humans and if IL-18 remains a risk factor after taking established clinical risk factors, CRP and IL6, into account.

In the present study, we used participants in the CV arm of the prospective population-based Malmö Diet and Cancer (MDC) study and the Surrogate Markers for Micro- and Macro-vascular Hard Endpoints for Innovative diabetes Tools Vascular Imaging Prediction (SUMMIT VIP) study to investigate whether IL-18 is associated with risk of MI and CV death, atherosclerosis severity, and vascular dysfunction. The relationship between IL-18 and risk of myocardial infarction and CV death was further validated and compared to IL-6 in the UK Biobank population.

## 2. Methods

### 2.1 Study cohorts

The MDC study is a prospective population-based cohort (*n* = 28 449) study examining the association between diet and cancer.^12^ Participants born between 1926 and 1945 living in Malmö, Sweden, were eligible for inclusion in the study. Between October 1991 and February 1994, every other participant was also invited to take part in a sub-study focusing on the epidemiology of carotid artery disease (MDC study CV arm, *n* = 6103).^13^ Out of these, 5405 came to a second examination where fasting plasma samples were collected. We excluded 545 of these participants from the present study due to incomplete clinical data. Additionally, 118 participants were excluded because the analysis of their plasma samples did not pass the internal quality control for the biomarker analyses. The remaining 4742 participants were followed from baseline examination until first myocardial infarction, emigration from Sweden, or death, up until 31 December 2020, using the Swedish Hospital Discharge Register and the National Cause of Death Register. Myocardial infarction was defined as a fatal or non-fatal myocardial infarction based on the International Classification of Diseases 9th and 10th revisions (ICD-9 and ICD-10) codes 410 and I21. Diabetes at baseline was based on baseline interviews and data from national registries. Risk factors and carotid intima–media thickness (IMT) were determined as previously described.^13^

The SUMMIT VIP baseline study investigation included 458 participants with Type 2 diabetes (T2D) and clinically manifest CVD, 527 participants with T2D but without clinical signs of CVD, 245 participants with CVD but without T2D, and 270 participants with neither CVD nor T2D. Participants were recruited from existing population cohorts and hospital registers at the university hospitals in Malmö (Sweden), Pisa (Italy), Dundee and Exeter (UK) between November 2010 and June 2013 as previously described.^14,15^ We excluded 62 participants from this study due to lack of biomarker data. All studies were approved by the respective regional ethical review boards and conducted in accordance with the Helsinki Declaration. All participants gave written informed consent. The reporting of the studies is in accordance with the STROBE guidelines (see Figure e1).

### 2.2 UK Biobank Plasma Proteomics Project

The UK Biobank Plasma Proteomics Project included 54 219 participants from the general UK population with plasma proteomics profiled with the Olink Explore assay.^16^ Incident fatal or non-fatal myocardial infarction and CV death were defined based on ICD-10 codes (see eMethods). IL-18 and IL-6 were measured as part of the Olink Explore 3072 panel. UK Biobank data were accessed under the application number 68601.

### 2.3 Proximity extension assay

Plasma levels of IL-6 and IL-18 in the MDC cohort and in the SUMMIT VIP study were analysed by the proximity extension assay (PEA) technique using the Proseek Multiplex CVD^96×96^ reagent kit (Olink Bioscience, Uppsala, Sweden) at the Clinical Biomarkers Facility, Science for Life Laboratory, Uppsala. The CV for intra-assay variation (within-run) and inter-assay variation (between-run) were 6 and 12% for IL-6 and 11 and 11% for IL-18, respectively, and the analytical ranges were 0.8–3125 pg/mL and 0.5–125 000 pg/mL, respectively. All data were presented as normalized protein expression values (NPX). The NPX units in the Proseek Multiplex CVD^96×96^ (MDC and SUMMIT) and Olink Explore (UK Biobank) assays are produced with different algorithms and have different numeric scales (log2 intensity for Proseek Multiplex CVD^96×96^ vs. median-centred log ratio of each patient sample to technical control samples for Explore). However, both assays offer relative quantification. In other words, the protein levels could be compared between participants within a cohort (higher vs. lower levels) but could not be compared between cohorts or translated into absolute concentration.

### 2.4 Vascular assessments in the SUMMIT VIP cohort

The IMT of the right and left common carotid artery (CCA) and the carotid bulbs were measured by ultrasound as previously described.^17^ Endothelial function was measured using an EndoPAT device (Itamar Medical, Caesarea Ind. Park, Israel) to estimate the endothelium-dependent post-ischaemic hyperaemia in response to 5 min of upper arm arterial occlusion. Arterial stiffness was assessed by calculating the carotid-femoral pulse wave velocity (PWV) using a SphygmoCor device (Atcor Medical, Australia).

### 2.5 Flow cytometry

Immunophenotyping of monocytes, T cells, and B cells in the MDC study was performed by flow cytometry as described previously.^18^ Briefly, mononuclear cells were isolated from blood collected at the baseline examination with density gradient centrifugation in Lymphoprep (Stemcell Technologies). To analyse CD3^+^CD4^+^, CD3^+^CD8^+^ T cells and regulatory T cells (CD3^+^CD4^+^CD25^+^FoxP3^+^), 4 × 10^5^ cells were stained with fluorochrome-labelled antibodies against CD3-PE/Cy7, CD4-PB, CD8-AF700, CD25-PE, and FoxP3-APC (all from BioLegend, except FoxP3 from eBioscience) for 30 min at 4°C. Before Foxp3 staining, cells were incubated and washed with Foxp3 Fixation/Permeabilization buffers (eBioscience). Cell viability was determined by 7-aminoactinomycin D (7AAD) staining, and stained cells were fixed in 1% paraformaldehyde. Th1 (CD3^+^CD4^+^IFN-γ^+^) and Th2 (CD3^+^CD4^+^IL-4^+^) cells were determined in samples stimulated with PMA (10 ng), ionomycin (0.2 µg), and brefeldin A (1 µg, all from Sigma) for 4 h at 37°C before staining with antibodies against CD3-PE/Cy7, CD4-PB, PE-anti-IFN-γ-PE, and IL-4-APC. To analyse classical (CD14^++^CD16^−^) and non-classical (CD14^+^CD16^++^) monocytes, samples were stained with CD14-Pacific Blue and CD16-PE/Cy7 antibodies, and B cells (CD19^+^) were stained with a CD19-FITC antibody. Flow cytometry data were acquired on an ADP-CyAn flow cytometer (Beckman Coulter), and analysis was performed using FlowJo 7.5.5 (Treestar Inc.). CompBeads (BD Biosciences) were used to correct for fluorescence spillover in multicolour analyses, and gate boundaries were set by fluorescence-minus-one (FMO) controls. Cell numbers in blood were calculated by multiplying percentages of gated monocyte populations with counts obtained from a blood cell count analysis, using a Sysmex K-1000 with data unit DA 1000 (TOA Medical Electronics Co.).

### 2.6 Statistical analysis

Analysis of skewness and kurtosis was used to test for normality. Values are given as mean and standard deviation (SD), median and interquartile range (IQR), or percentages as appropriate. Differences between means of normally distributed continuous variables were assessed using independent sample *t*-tests and between skewed variables using the Mann–Whitney *U* test. The *χ*^2^ test was used for categorical variables. Correlation coefficients between continuous variables were calculated using the Spearman rank test. The relation between biomarker tertiles and risk of myocardial infarction or CV death during follow-up was assessed by Cox proportional hazards regression, and Kaplan–Meier survival curves were used for visualization. The time to event or censoring was the time from baseline investigation to the first occurrence of myocardial infarction, CV death, or end of follow-up which was 31 December 2020 (administrative censoring). IBM SPSS Statistics 29, R v.4.5.0, and R Studio v.2024.12.1 (using the packages survival, survminer, and ggplot2) were used for statistical analyses.

Statistical analysis of the UK Biobank data was methodologically similar and performed in Python and R as described in eMethods.^19^

## 3. Results

### 3.1 IL-18 and CV risk in the MDC study

The MDC study population consisted of 1895 men and 2847 women with a mean age of 57.5 ± 6.0 years at baseline. During a mean follow-up of 23.0 ± 7.1 years, 617 participants suffered a fatal or non-fatal MI, and 644 participants died of CVD. Participants with incident MI were older and were more often men, and there was a general overrepresentation of CV risk factors in this group. They also had higher baseline levels of IL-18 and hsCRP (*Table 1*). Both inflammatory markers showed significant associations with age; metabolic risk factors, such as low high-density lipoproteins (HDL) cholesterol and high triglycerides; and blood pressure. IL-18 was moderately associated with hsCRP (see Figure e2). IL-18 was higher in men than in women (10.1 ± 0.60 vs. 9.81 ± 0.61 NPX, *P* < 0.001), in participants with diabetes than participants without diabetes (10.2 ± 0.64 vs. 9.90 ± 0.62 NPX, *P* < 0.001), and in current smokers vs. non- and former smokers (10.0 ± 0.60 vs. 9.90 ± 0.63 NPX, *P* < 0.001).

**Table 1:** Baseline clinical characteristics of study participants with and without incident myocardial infarction in the MDC cohort

	No myocardial infarction (*n* = 4125)	Incident myocardial infarction (*n* = 617)
Age (years)	57.2 ± 6.0	59.5 ± 5.4***
Sex [*n* (%) males]	1542 (37.4)	353 (57.2)***
Smoking [*n* (%)]	868 (21.1)	152 (26.7)*
Diabetes [*n* (%)]	149 (3.6)	52 (8.4)***
BMI (kg/m^2^)	25.5 ± 3.9	26.6 ± 4.2***
HbA1c (%)	4.86 ± 0.66	5.07 ± 0.96***
LDL (mmol/L)	4.15 ± 0.98	4.30 ± 0.92***
HDL (mmol/L)	1.41 ± 0.37	1.27 ± 0.35***
TG (mmol/L)	1.13 (0.85–1.55)	1.29 (0.95–1.74)***
Systolic BP (mmHg)	140 ± 19	148 ± 19***
Diastolic BP (mmHg)	86 ± 9	89 ± 10***
eGFR (mL/min/1.73m^2^)	74.7 ± 14.6	75.2 ± 14.6
hsCRP (mg/L)	1.30 (0.60–2.70)	1.60 (0.80–3.30)***
IL-6 (NPX)	4.36 ± 1.03	4.68 ± 0.95***
IL-18 (NPX)	9.91 ± 0.62	10.03 ± 0.61***

Variables with normal distribution are shown as mean and SD, and statistical differences between groups are calculated by Student’s *t*-test. Variables with skewed distribution are shown as median and IQR, and statistical differences between groups are calculated by the Mann–Whitney *U* test. Statistical differences between categorical variables were analysed by the *χ*^2^ test. BMI, body mass index; f-glucose, fasting glucose; HbA1c, haemoglobin A1c; LDL, low-density lipoproteins; HDL, high-density lipoproteins; TG, triglycerides; hsCRP, high-sensitivity C-reactive protein; NPX, normalized protein expression.

^*^ *P* < 0.05.

^***^ *P* < 0.001 as compared with those without event.

**Figure 1:**
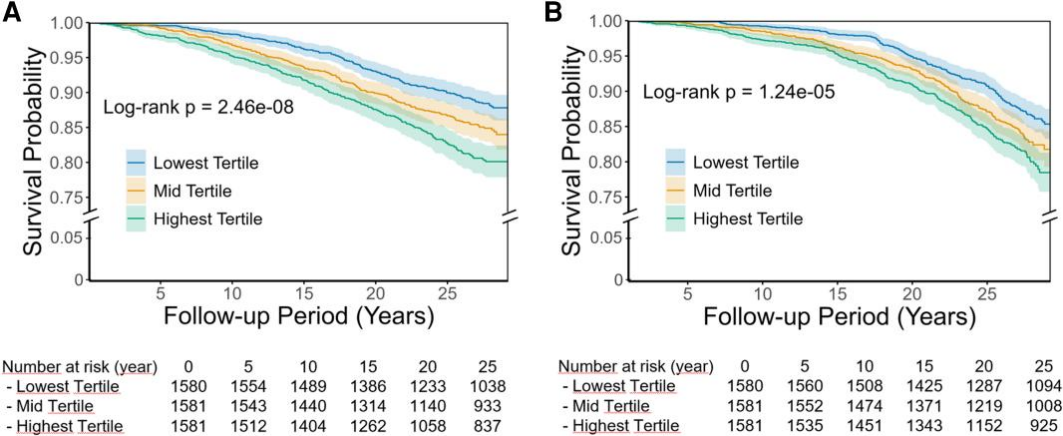
Associations of IL-18 with risk of myocardial infarction and CV death in the MDC cohort. Kaplan–Meier plots demonstrating the association between IL-18 tertiles with risk of (*A*) myocardial infarction and (*B*) CV death. Significance of differences was calculated using the log-rank test. Lowest tertile 6.731–9.657 NPX, mid tertile 9.658–10.175 NPX, highest tertile 10.175–12.689 NPX.

**Figure 2:**
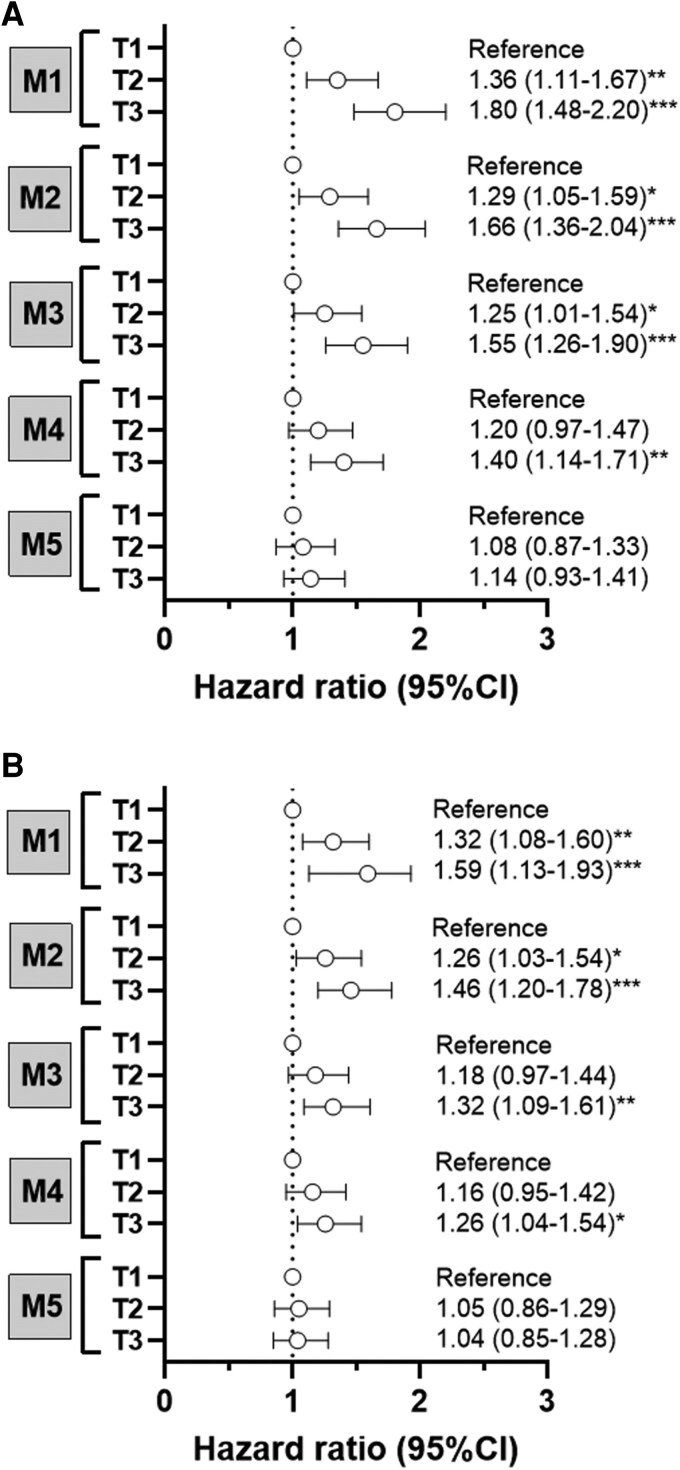
Forest plots summarizing associations between IL-18 tertiles and myocardial infarction or CV death in the MDC cohort. Cox proportional hazard regression models (M) were used to determine myocardial infarction (*A*) or CV death (*B*). Hazard ratios (HRs) are shown for the 2nd (T2) or 3rd (T3) tertile relative to the 1st reference tertile (T1) of the plasma levels of IL-18 in the MDC cohort (*n* = 4742). Models were adjusted as follows: M1 was unadjusted, M2 was adjusted for hsCRP, M3 was adjusted for IL-6, M4 was adjusted for age and sex, and M5 was adjusted for age, sex, total and HDL cholesterol, systolic blood pressure, diabetes, current smoking, and previous myocardial infarction. *P < 0.05, **P < 0.01 and ***P < 0.001.

Participants in the highest tertile of IL-18 had the highest risk of MI and CV death (*Figure 1*). When adjusting for hsCRP or for IL-6 in Cox proportional hazards regression models, the highest IL-18 tertile remained significantly associated with increased risk of both MI and CV death. Similar associations were found when adjusting for age and sex, whereas they were lost when also adjusting for total and HDL cholesterol, systolic blood pressure, diabetes, current smoking, and prior myocardial infarction (*Figure 2*). However, if HDL was excluded from the latter model, IL-18 remained statistically associated with risk of MI (*P* = 0.03). The association of IL-6 with CV risk in the MDC cohort has been previously reported.^20^

### 3.2 IL-18 and CV risk in the UK Biobank

Association of IL-18 with risk of MI that is lost following adjustment for clinical risk factors has been observed in two earlier population cohorts of sizes comparable to that of the MDC cohort.^21,22^ To explore if an independent association between IL-18 and MI could be demonstrated in a larger cohort, we used data available for 54 219 participants in the UK Biobank Plasma Proteomics Project (see Tables e1 and e2). The baseline characteristics of the UK Biobank Plasma Proteomics Project were overall similar to MDC, including a similar distribution of hsCRP levels. The UK Biobank participants were slightly more often male (e.g. 45% vs. 37% among patients without incident myocardial infarction) and less often smoking (e.g. 11% vs. 21% among patients without incident myocardial infarction) compared to the MDC cohort. The UK Biobank participants who experienced MI or CV death during the 15 years of follow-up were more likely to be older, male, and smokers, have diabetes at baseline, and have a history of prior myocardial infarction within 5 years before the baseline assessment (see Tables e1 and e2). They also had higher levels of hsCRP, systolic blood pressure, and triglycerides at baseline (see Tables e1 and e2). IL-6, IL-18, and hsCRP showed weak to moderate (i.e. 0.1–0.4 range) positive correlation with BMI, HbA1c, blood pressure, and triglycerides at baseline (see Figure e3).

**Figure 3:**
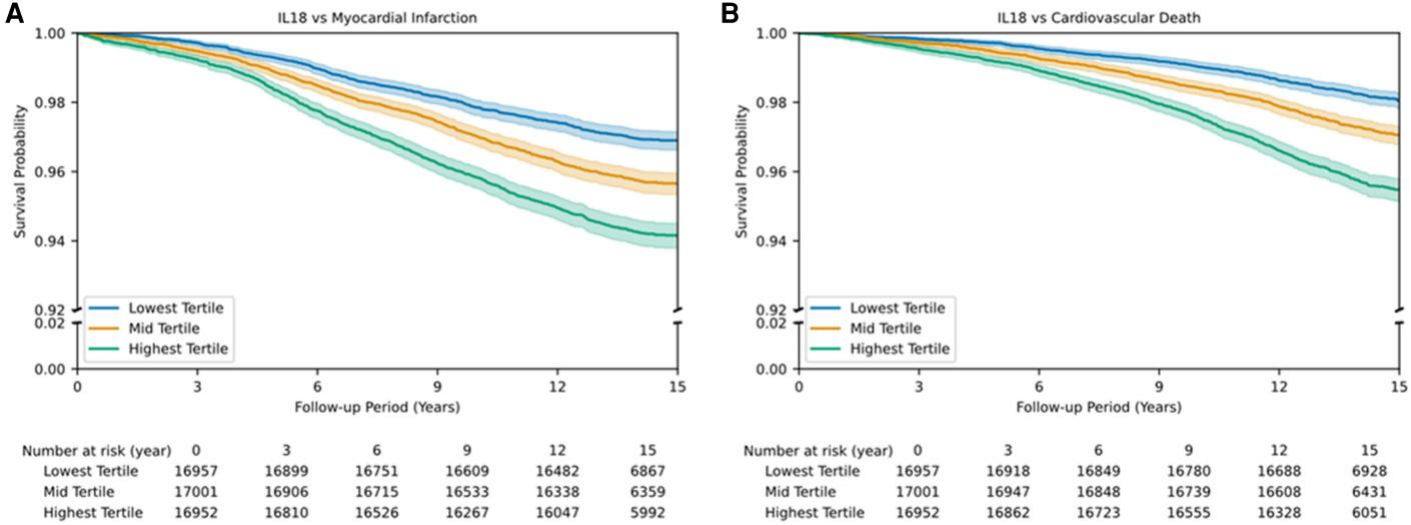
Associations of IL-18 with risk of myocardial infarction and CV death in the UK Biobank cohort. Kaplan–Meier plots demonstrating the association between IL-18 tertiles with risk of (*A*) myocardial infarction and (*B*) CV death. Significance of differences was calculated using the log-rank test. Lowest tertile less than −0.432 NPX, mid tertile greater than or equal to −0.432 to 0.432 NPX, highest tertile >0.432 NPX. Note that the NPX values generated by Olink Explore for the UK Biobank samples are on a different numeric scale than those generated by Olink Proseek Multiplex for MDC samples.

**Figure 4:**
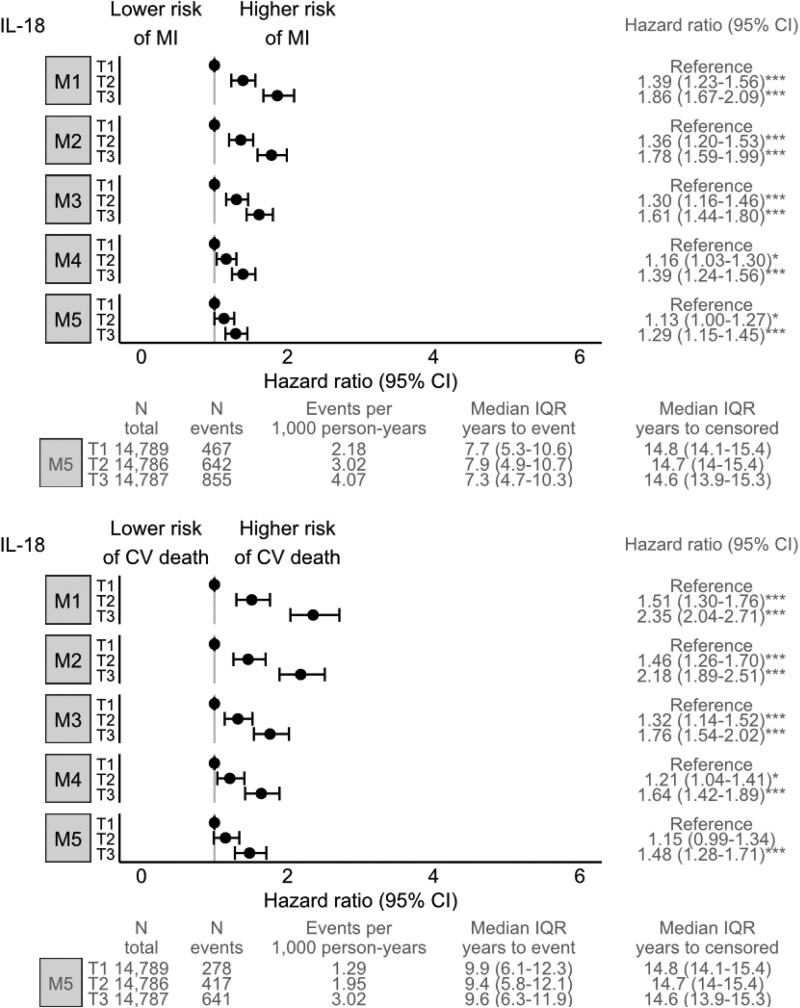
Forest plots summarizing associations between IL-18 tertiles and myocardial infarction or CV death in the UK Biobank cohort. Cox proportional hazard regression models (M) were adjusted as follows: M1 was unadjusted; M2 was adjusted for hsCRP; M3 was adjusted for IL-6; M4 was adjusted for age and sex; M5 was adjusted for age, sex, non-HDL cholesterol, systolic blood pressure, diabetes, current smoking, and prior myocardial infarction. *P < 0.05, **P < 0.01 and ***P < 0.001. T1, lowest tertile; T2, middle tertile; T3, highest tertile. Models M1, M2, M4, and M5 were estimated on the subset of participants who had all M5 covariate measurements available at baseline. M3 was estimated on a larger set of all participants with available Olink IL-6 and IL-18 measurements at baseline.

Participants with elevated IL-6 and IL-18 at baseline were at increased risk of incident MI and CV death in the UK Biobank (see Figures e4 and e5, *Figure 3*). The associations between IL-6 and IL-18 and the CV outcomes remained significant after full covariate adjustment, including established clinical risk factors and inflammation markers (see Figure e6, *Figure 4*). IL-6 had higher hazard ratios than IL-18 for both CV death and myocardial infarction, and both as a standalone risk factor and after full covariate adjustment. IL-6 and IL-18 were not redundant with any single clinical risk factor, considered separately. However, we observed that participants with a higher overall burden of clinical risk factors had elevated IL-6 and IL-18 at baseline. Therefore, addition of IL-6 and IL-18 resulted in a different numeric description of the overall risk (representative example Figure e7) but did not improve risk stratification beyond the clinical risk factors (see Table e3).

### 3.3 Associations of IL-18 with atherosclerosis and vascular function in SUMMIT VIP

**Figure 5:**
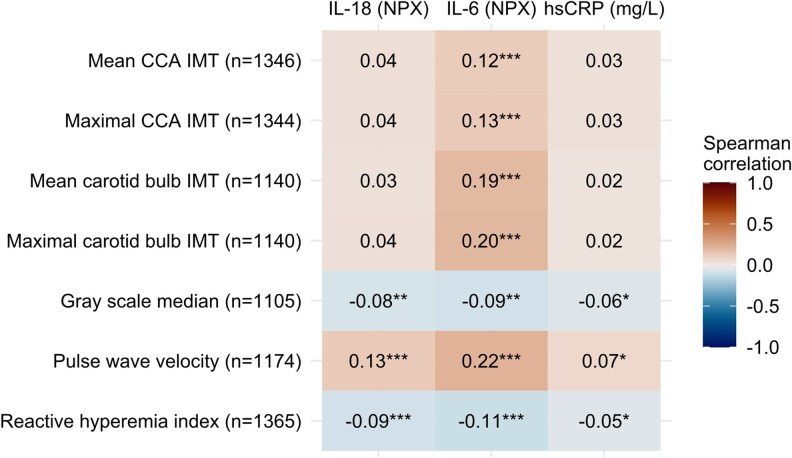
Associations of IL-18, IL-6, and hsCRP with markers of atherosclerosis and vascular function in the SUMMIT VIP cohort. CCA, common carotid artery; IMT, intima–media thickness; ABPI, ankle brachial pressure index. Correlation strengths are defined by the Spearman rank correlation coefficients. *P*-values are shown. *P < 0.05, **P < 0.01, ***P < 0.001.

To further investigate the associations of IL-18 with the severity of atherosclerosis, as well as with arterial stiffness and endothelial function in participants with more advanced CVD, we used data from the SUMMIT VIP study.^14^ This cohort included 985 participants with Type 2 diabetes and 515 participants without diabetes. Approximately half of the participants had a history of CV disease, including MI, stroke, or peripheral artery disease. Accordingly, the participants in this cohort could be anticipated to have more advanced arterial disease than those in the MDC cohort and would thus be more appropriate for studies of the associations of IL-18, IL-6, and hsCRP with atherosclerosis severity and functional arterial changes than the MDC population cohort. Ultrasound analyses of the IMT in the CCA and the carotid bulb were used as surrogate markers of atherosclerosis severity. Plasma levels of IL-6 correlated significantly with both mean and maximal CCA and carotid bulb IMT (*Figure 5*), and these associations remained statistically significant after adjusting for age, sex, and the presence of diabetes and history of CV disease in linear regression models. In contrast, there were no significant associations of IL-18 and hsCRP with carotid IMT. Analysing the greyscale median of atherosclerotic plaques provides information about plaque structure with a high greyscale median reflecting a more fibrous tissue content.^23^ IL-18 and hsCRP were both inversely associated with the greyscale median of plaques detected in the carotid bulb indicating the presence of less stable plaques (*Figure 3*). Arterial ageing leads to stiffening of arteries, a phenomenon that can be analysed by determining the PWV and that is associated with increased CV risk.^24^ Higher levels of IL-18 and hsCRP were both weakly associated with increased PWV (*Figure 5*). We also found that high levels of both biomarkers were weakly associated with impaired endothelial function as assessed by endothelium-dependent vasodilation in response to post-ischaemic hyperaemia using the EndoPAT technology (see Table e3). The associations of IL-18 and hsCRP with PWV as well as the association between IL-18 and endothelial function remained statistically significant after adjusting for age, sex, and the presence of diabetes and history of CV disease in linear regression models, while the statistical associations of IL-18 and hsCRP with plaque greyscale median as well as the association between hsCRP and endothelial function were lost.

### 3.4 Associations of IL-18, IL-6, and hsCRP with immune cell subsets

**Figure 6:**
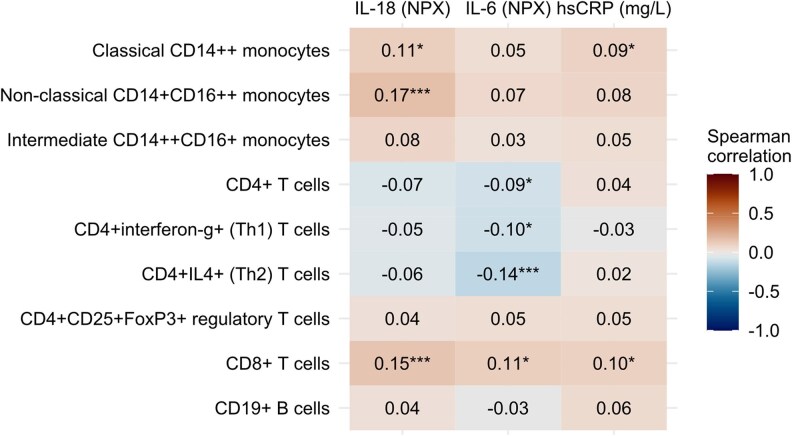
Associations of IL-18, IL-6, and hsCRP with immune cell subsets in the MDC cohort. Correlation strengths are defined by the Spearman rank correlation coefficients. *P*-values are shown. *P < 0.05 and ***P < 0.001.

Both innate and adaptive immune cells are known to play important roles in atherosclerosis.^25^ IL-18 regulates the function of many types of immune cells but is particularly important for the activation of interferon-γ producing Th1 cells and CD8^+^ T cells.^26^ In a subset of 527 randomly selected participants from the MDC study, we determined the associations of IL-18 and hsCRP with several peripheral blood mononuclear immune cell subsets. High levels of IL-18 were associated with elevated numbers of classical CD14^++^ and non-classical CD14^+^CD16^++^ monocytes as well as with elevated number of CD8^+^ T cells (*Figure 6*). IL-18 was not associated with the numbers of regulatory T cells, B cells, or CD4^+^interferon-γ^+^ Th1 cells. The associations of hsCRP with immune cell subsets were generally weaker than those of IL-18 (*Figure 6*). Higher IL-6 was associated with CD8^+^ T cells but weaker than that for IL-18. IL-6 was also inversely associated with CD4+ T cells (helper T cells), Th1 and Th2 cell types (*Figure 6*).

## 4. Discussion

The role of NLRP3 inflammasome activation in the development and destabilization of atherosclerotic lesions has attracted increasing interest during recent years.^27^ Several therapies targeting the inflammasome directly or by blocking the cytokines released upon inflammasome activation have been or are currently being clinically studied.^9^ Of the cytokines released as a result of inflammasome activation, there has been most focus on IL-1β/IL-6, while IL-18 has received less attention. In the present study, we determined the association of IL-18 with the risk of MI and CV death in middle-aged men and women from the MDC study and UK Biobank. We also analysed the associations of IL-18 with atherosclerosis severity and arterial function in the high-risk population of the SUMMIT VIP study. Our findings show that participants with elevated levels of IL-18 have an increased risk of MI and CV death. IL-18 levels were associated with well-established CV risk factors such as age, male sex, smoking, high blood pressure, and diabetes. When adjusting for these potential confounders, IL-18 remained independently associated with the risk for MI and CV death in the UK Biobank cohort, but not in the smaller MDC cohort.

The association of IL-18 with risk of MI has previously been investigated in two population cohorts. In the Prospective Epidemiological Study of Myocardial Infarction (PRIME) study that analysed 335 cases with a coronary event to 670 age-matched controls in a nested case-control manner, Blankenberg and coworkers^21^ found that baseline levels of IL-18 were significantly higher in men who developed a coronary event than in controls. Coronary events included non-fatal MI, coronary death, and angina pectoris. However, after adjusting for confounders only incident angina, but not risk of MI and coronary death, remained independently associated with IL-18. Similar observations were recently made in a report from the Atherosclerosis Risk in Communities (ARIC) study in which the association between IL-18 and risk of MI was lost after adjusting for confounders.^22^ Hence, the observations made in the present study of the UK Biobank cohort are, to the best of our knowledge, the first to identify elevated IL-18 as an independent risk factor of myocardial infarction in a general population setting.

We performed an unpowered exploratory analysis in the Malmö Cancer and Diet study and UK Biobank. Our sample size was based on the availability of Olink proteomics measurements, which is a limitation of our research. We performed sample size calculation for future studies (see Table e4), and it indicated that much larger cohorts would be needed to detect the association between the risk of CV events and IL-18 than IL-6, both in the general population and in patient population with as high event rates as in the MDC cohort. This potentially explains why IL-6 is more commonly reported in literature as a CV risk factor compared to IL-18,^22,28,29^ and why the association between IL-18 and CV risk held after full covariate adjustment in the larger UK Biobank cohort but not in the MDC cohort.

We observed that IL-18 and IL-6 were associated with CV risk but did not add value as risk stratification biomarkers beyond the established clinical risk factors. Lack of or very small added value for risk stratification appears to be a common issue for protein biomarkers^30^ and is not limited to IL-18 and IL-6. However, we believe that the association with CV risk is interesting in itself because elevated IL-6 and IL-18 seemed to indicate overall ‘sicker’ participants a decade or, in some cases, even earlier before they experienced an event. Elevation of IL-6 or IL-18 might be driven by other factors such as older age or diabetes. However, IL-6 and IL-18 represent risk factors that are potentially modifiable with pharmacological agents. Clinical evidence from the IL-1β blocking antibody canakinumab in the CANTOS trial suggests that lowering inflammatory tone might lower CV risk.^31^ Canakinumab significantly reduced CRP and IL-6.^31,32^ However, canakinumab did not lower IL-18 levels, and higher on-treatment levels of IL-6 and IL-18 were still associated with residual CV risk.^32^ Inhibition of IL-18 with recombinant IL-18 binding protein showed protection against atherosclerosis^10^ whereas administration of IL-18 aggravated atherosclerosis^11^ in mice. The translational relevance of IL-18 reduction for protection against CV events is supported by a recent Mendelian randomization study in the UK Biobank.^33^ Another Mendelian randomization study based on data from the Scallop consortium found evidence for a functional role of IL-18 in peripheral artery disease and cardiometabolic disease.^33^ In humans IL-18 is expressed in macrophage-rich regions of atherosclerotic plaques, and IL-18 mRNA levels are increased in plaques associated with acute cerebrovascular events as compared with asymptomatic carotid plaques.^34,35^ Drugs specifically blocking IL-18 signalling are in early stages of clinical development and have not been evaluated for their impact on CV risk.^36^ Thus, clinical studies informing of the efficacy of IL-6, IL-18, or both IL-6 and IL-18 for CV event prevention remain to be seen.

There have been reports of association between IL-18 and carotid IMT in smaller cohorts,^37,38^ but we could not replicate this in the present study. Both IL-18 and hsCRP showed modest inverse associations with carotid plaque greyscale median and reactive hyperaemia index in response to post-ischaemic hyperaemia indicating that high levels were associated with less stable plaques and impaired endothelial function.

Although the precise role of IL-18 in the immune system remains to be fully elucidated, it has been shown to be of importance for activation of CD8^+^ T cells.^26^ We found a significant association between IL-18 and the number of CD8^+^ T cells when analysing peripheral blood mononuclear cells isolated at baseline from participants participating in the MDC. IL-18-dependent activation of CD8^+^ T cells may increase the risk of coronary events since cytotoxic CD8^+^ T cells have been identified as one factor contributing to plaque cell death and necrotic core progression.^39^

There are some limitations of the present study that need to be considered. Importantly, significant association of IL-18 with CVD risk after adjustment for clinical risk factors was observed in the secondary but not the primary cohort. IL-6, IL-18, and hsCRP levels were also only determined at the baseline investigation, and we lack data on changes in plasma levels during the follow-up period. Moreover, we did not have data on changes in medication during follow-up. Finally, it should be kept in mind that observations made in general population cohorts such as MDC and UK Biobank cannot be directly extrapolated to high-risk populations such as subjects with chronic coronary syndromes.

In conclusion, our prospective studies show that participants with elevated levels of IL-18 have an increased risk of MI and CV death. However, IL-18 did not add value as risk stratification biomarker beyond the established clinical risk factors. IL-18 elevation is associated with major CV risk factors, and taken together with results from previous experimental studies, these observations support the notion that IL-18 is involved in the atherosclerotic disease process. Targeting IL-18 directly or through inflammasome inhibition represents a promising approach for treatment of residual inflammatory risk in high-risk atherosclerosis patients that merits to be evaluated in future randomized clinical trials.

Translational perspective This study shows that elevated IL-18 is associated with an increased risk of myocardial infarction and CV death and is a non-redundant risk factor with CRP and IL-6 in the general population. Targeting IL-18 directly or through inflammasome inhibition represents a promising approach for treatment of residual inflammatory risk in high-risk atherosclerosis patients that merits to be evaluated in future randomized clinical trials.

## Supplementary Material

cvag135_Supplementary_Data

## Data Availability

MDC data can be accessed upon application to the MDC Steering Committee (https://www.malmo-kohorter.lu.se). Restrictions may apply. UK Biobank data should be requested following the application process https://www.ukbiobank.ac.uk/enable-your-research/apply-for-access.

## References

[1] Ridker PM . Targeting residual inflammatory risk: the next frontier for atherosclerosis treatment and prevention. Vascul Pharmacol 2023;153:. 10.1016/j.vph.2023.107238 37871757

[2] Ridker PM, Everett BM, Thuren T, MacFadyen JG, Chang WH, Ballantyne C, Fonseca F, Nicolau J, Koenig W, Anker SD, Kastelein JJP, Cornel JH, Pais P, Pella D, Genest J, Cifkova R, Lorenzatti A, Forster T, Kobalava Z, Vida-Simiti L, Flather M, Shimokawa H, Ogawa H, Dellborg M, Rossi PRF, Troquay RPT, Libby P, Glynn RJ, Group CT. Antiinflammatory therapy with canakinumab for atherosclerotic disease. N Engl J Med 2017;377:1119–1131. 28845751 10.1056/NEJMoa1707914

[3] Tardif JC, Kouz S, Waters DD, Bertrand OF, Diaz R, Maggioni AP, Pinto FJ, Ibrahim R, Gamra H, Kiwan GS, Berry C, Lopez-Sendon J, Ostadal P, Koenig W, Angoulvant D, Gregoire JC, Lavoie MA, Dube MP, Rhainds D, Provencher M, Blondeau L, Orfanos A, L'Allier PL, Guertin MC, Roubille F. Efficacy and safety of low-dose colchicine after myocardial infarction. N Engl J Med 2019;381:2497–2505. 31733140 10.1056/NEJMoa1912388

[4] Nidorf SM, Fiolet ATL, Mosterd A, Eikelboom JW, Schut A, Opstal TSJ, The SHK, Xu XF, Ireland MA, Lenderink T, Latchem D, Hoogslag P, Jerzewski A, Nierop P, Whelan A, Hendriks R, Swart H, Schaap J, Kuijper AFM, van Hessen MWJ, Saklani P, Tan I, Thompson AG, Morton A, Judkins C, Bax WA, Dirksen M, Alings M, Hankey GJ, Budgeon CA, Tijssen JGP, Cornel JH, Thompson PL. Lodoco2 trial I. Colchicine in patients with chronic coronary disease. N Engl J Med 2020;383:1838–1847. 32865380 10.1056/NEJMoa2021372

[5] Broz P, Dixit VM. Inflammasomes: mechanism of assembly, regulation and signalling. Nat Rev Immunol 2016;16:407–420. 27291964 10.1038/nri.2016.58

[6] Ridker PM . From C-reactive protein to interleukin-6 to interleukin-1: moving upstream to identify novel targets for atheroprotection. Circ Res 2016;118:145–156. 26837745 10.1161/CIRCRESAHA.115.306656 PMC4793711

[7] Ridker PM, Rane M. Interleukin-6 signaling and anti-interleukin-6 therapeutics in cardiovascular disease. Circ Res 2021;128:1728–1746. 33998272 10.1161/CIRCRESAHA.121.319077

[8] Nordestgaard BG . Does elevated C-reactive protein cause human atherothrombosis? Novel insights from genetics, intervention trials, and elsewhere. Curr Opin Lipidol 2009;20:393–401. 19667982 10.1097/MOL.0b013e3283307bfe

[9] Toldo S, Mezzaroma E, Buckley LF, Potere N, Di Nisio M, Biondi-Zoccai G, Van Tassell BW, Abbate A. Targeting the NLRP3 inflammasome in cardiovascular diseases. Pharmacol Ther 2022;236:108053. 34906598 10.1016/j.pharmthera.2021.108053 PMC9187780

[10] Mallat Z, Corbaz A, Scoazec A, Graber P, Alouani S, Esposito B, Humbert Y, Chvatchko Y, Tedgui A. Interleukin-18/interleukin-18 binding protein signaling modulates atherosclerotic lesion development and stability. Circ Res 2001;89:E41–E45. 11577031 10.1161/hh1901.098735

[11] Whitman SC, Ravisankar P, Daugherty A. Interleukin-18 enhances atherosclerosis in apolipoprotein E(-/-) mice through release of interferon-gamma. Circ Res 2002;90:E34–E38. 11834721 10.1161/hh0202.105292

[12] Berglund G, Elmstahl S, Janzon L, Larsson SA. The Malmo Diet and Cancer Study. Design and feasibility. J Intern Med 1993;233:45–51. 8429286 10.1111/j.1365-2796.1993.tb00647.x

[13] Hedblad B, Nilsson P, Janzon L, Berglund G. Relation between insulin resistance and carotid intima-media thickness and stenosis in non-diabetic subjects. Results from a cross-sectional study in Malmo, Sweden. Diabet Med 2000;17:299–307. 10821297 10.1046/j.1464-5491.2000.00280.x

[14] Shore AC, Colhoun HM, Natali A, Palombo C, Ostling G, Aizawa K, Kennback C, Casanova F, Persson M, Gooding K, Gates PE, Khan F, Looker HC, Adams F, Belch J, Pinnoli S, Venturi E, Morizzo C, Goncalves I, Ladenvall C, Nilsson J; SUMMIT consortium. Measures of atherosclerotic burden are associated with clinically manifest cardiovascular disease in type 2 diabetes: a European cross-sectional study. J Intern Med 2015;278:291–302. 25752315 10.1111/joim.12359

[15] Khan F, Goncalves I, Shore AC, Natali A, Palombo C, Colhoun HM, Ostling G, Casanova F, Kennback C, Aizawa K, Persson M, Gooding KM, Strain D, Looker H, Dove F, Belch J, Pinnola S, Venturi E, Kozakova M, Nilsson J. Plaque characteristics and biomarkers predicting regression and progression of carotid atherosclerosis. Cell Rep Med 2022;3:100676. 35858591 10.1016/j.xcrm.2022.100676 PMC9381367

[16] Sun BB, Chiou J, Traylor M, Benner C, Hsu YH, Richardson TG, Surendran P, Mahajan A, Robins C, Vasquez-Grinnell SG, Hou L, Kvikstad EM, Burren OS, Davitte J, Ferber KL, Gillies CE, Hedman ÅK, Hu S, Lin T, Mikkilineni R, Pendergrass RK, Pickering C, Prins B, Baird D, Chen CY, Ward LD, Deaton AM, Welsh S, Willis CM, Lehner N, Arnold M, Wörheide MA, Suhre K, Kastenmüller G, Sethi A, Cule M, Raj A; Alnylam Human Genetics; AstraZeneca Genomics Initiative; Biogen Biobank Team; Bristol Myers Squibb; Genentech Human Genetics; GlaxoSmithKline Genomic Sciences; Pfizer Integrative Biology; Population Analytics of Janssen Data Sciences; Regeneron Genetics Center; Burkitt-Gray L, Melamud E, Black MH, Fauman EB, Howson JMM, Kang HM, McCarthy MI, Nioi P, Petrovski S, Scott RA, Smith EN, Szalma S, Waterworth DM, Mitnaul LJ, Szustakowski JD, Gibson BW, Miller MR, Whelan CD. Plasma proteomic associations with genetics and health in the UK Biobank. Nature 2023;622:329–338. 37794186 10.1038/s41586-023-06592-6 PMC10567551

[17] Goncalves I, Lindholm MW, Pedro LM, Dias N, Fernandes e Fernandes J, Fredrikson GN, Nilsson J, Moses J, Ares MP. Elastin and calcium rather than collagen or lipid content are associated with echogenicity of human carotid plaques. Stroke 2004;35:2795–2800. 15514195 10.1161/01.STR.0000147038.12073.59

[18] Tomas L, Bengtsson E, Andersson L, Badn W, Tengryd C, Persson A, Edsfeldt A, Nilsson PM, Schiopu A, Nilsson J, Goncalves I, Bjorkbacka H. Low levels of CD4(+)CD28(null) T cells at baseline are associated with first-time coronary events in a prospective population-based case-control cohort. Arterioscler Thromb Vasc Biol 2020;40:426–436. 31801374 10.1161/ATVBAHA.119.313032

[19] Davidson-Pilon C . Lifelines: survival analysis in Python. J Open Source Softw 2019;4:1317.

[20] Edsfeldt A, Goncalves I, Vigren I, Jovanovic A, Engstrom G, Shore AC, Natali A, Khan F, Nilsson J. Circulating soluble IL-6 receptor associates with plaque inflammation but not with atherosclerosis severity and cardiovascular risk. Vascul Pharmacol 2023;152:107214. 37634789 10.1016/j.vph.2023.107214

[21] Blankenberg S, Luc G, Ducimetiere P, Arveiler D, Ferrieres J, Amouyel P, Evans A, Cambien F, Tiret L, Group PS. Interleukin-18 and the risk of coronary heart disease in European men: the Prospective Epidemiological Study of Myocardial Infarction (PRIME). Circulation 2003;108:2453–2459. 14581397 10.1161/01.CIR.0000099509.76044.A2

[22] Jia X, Buckley L, Sun C, Rifai A, Yu M, Nambi B, Virani V, Selvin SS, Matsushita E, Hoogeveen K, Coresh RC, Shah J, Ballantyne AM, M C. Association of interleukin-6 and interleukin-18 with cardiovascular disease in older adults: atherosclerosis risk in communities study. Eur J Prev Cardiol 2023;30:1731–1740. 37306504 10.1093/eurjpc/zwad197 PMC10637765

[23] Gronholdt ML, Nordestgaard BG, Schroeder TV, Vorstrup S, Sillesen H. Ultrasonic echolucent carotid plaques predict future strokes. Circulation 2001;104:68–73. 11435340 10.1161/hc2601.091704

[24] Chirinos JA, Segers P, Hughes T, Townsend R. Large-artery stiffness in health and disease: JACC State-of-the-Art Review. J Am Coll Cardiol 2019;74:1237–1263. 31466622 10.1016/j.jacc.2019.07.012 PMC6719727

[25] Libby P, Lichtman AH, Hansson GK. Immune effector mechanisms implicated in atherosclerosis: from mice to humans. Immunity 2013;38:1092–1104. 23809160 10.1016/j.immuni.2013.06.009 PMC3764500

[26] Landy E, Carol H, Ring A, Canna S. Biological and clinical roles of IL-18 in inflammatory diseases. Nat Rev Rheumatol 2024;20:33–47. 38081945 10.1038/s41584-023-01053-w PMC12337794

[27] Khair M, Khair M, Vangaveti VN, Malabu UH. The role of the NLRP3 inflammasome in atherosclerotic disease: systematic review and meta-analysis. J Cardiol 2024;84:14–21. 38521117 10.1016/j.jjcc.2024.03.003

[28] Mehta NN, deGoma E, Shapiro MD. IL-6 and cardiovascular risk: a narrative review. Curr Atheroscler Rep 2024;27:12. 39589436 10.1007/s11883-024-01259-7 PMC11599326

[29] Jefferis BJ, Papacosta O, Owen CG, Wannamethee SG, Humphries SE, Woodward M, Lennon LT, Thomson A, Welsh P, Rumley A, Lowe GD, Whincup PH. Interleukin 18 and coronary heart disease: prospective study and systematic review. Atherosclerosis 2011;217:227–233. 21481392 10.1016/j.atherosclerosis.2011.03.015 PMC3146704

[30] Royer P, Bjornson E, Adiels M, Josefson R, Hagberg E, Gummesson A, Bergstrom G. Large-scale plasma proteomics in the UK Biobank modestly improves prediction of major cardiovascular events in a population without previous cardiovascular disease. Eur J Prev Cardiol 2024;31:1681–1689. 38546334 10.1093/eurjpc/zwae124

[31] Ridker PM, MacFadyen JG, Everett BM, Libby P, Thuren T, Glynn RJ, Group CT. Relationship of C-reactive protein reduction to cardiovascular event reduction following treatment with canakinumab: a secondary analysis from the CANTOS randomised controlled trial. Lancet 2018;391:319–328. 29146124 10.1016/S0140-6736(17)32814-3

[32] Ridker PM, MacFadyen JG, Thuren T, Libby P. Residual inflammatory risk associated with interleukin-18 and interleukin-6 after successful interleukin-1beta inhibition with canakinumab: further rationale for the development of targeted anti-cytokine therapies for the treatment of atherothrombosis. Eur Heart J 2020;41:2153–2163. 31504417 10.1093/eurheartj/ehz542

[33] Brennan SO, Kelly PJ, Gorey S, Synnott P, Gill D, Dichgans M, Georgakis MK, Dib MJ, Gagnon E, Mahon N, Blake GJ, Jern C, Markus HS, Whiteley W, McCabe JJ. Genetically predicted IL-18 inhibition and risk of cardiovascular events: a Mendelian randomization study. Circulation 2025;151:334–336. 39899632 10.1161/CIRCULATIONAHA.124.072238

[34] Mallat Z, Corbaz A, Scoazec A, Besnard S, Leseche G, Chvatchko Y, Tedgui A. Expression of interleukin-18 in human atherosclerotic plaques and relation to plaque instability. Circulation 2001;104:1598–1603. 11581135 10.1161/hc3901.096721

[35] Paramel Varghese G, Folkersen L, Strawbridge RJ, Halvorsen B, Yndestad A, Ranheim T, Krohg-Sorensen K, Skjelland M, Espevik T, Aukrust P, Lengquist M, Hedin U, Jansson JH, Fransen K, Hansson GK, Eriksson P, Sirsjo A. NLRP3 inflammasome expression and activation in human atherosclerosis. J Am Heart Assoc 2016;5:e003031. 27207962 10.1161/JAHA.115.003031 PMC4889178

[36] Novick D . IL-18 and IL-18BP: a unique dyad in health and disease. Int J Mol Sci 2024;25:13505. 39769266 10.3390/ijms252413505 PMC11727785

[37] Yamagami H, Kitagawa K, Hoshi T, Furukado S, Hougaku H, Nagai Y, Hori M. Associations of serum IL-18 levels with carotid intima-media thickness. Arterioscler Thromb Vasc Biol 2005;25:1458–1462. 15860738 10.1161/01.ATV.0000168417.52486.56

[38] Nakamura A, Shikata K, Hiramatsu M, Nakatou T, Kitamura T, Wada J, Itoshima T, Makino H. Serum interleukin-18 levels are associated with nephropathy and atherosclerosis in Japanese patients with type 2 diabetes. Diabetes Care 2005;28:2890–2895. 16306550 10.2337/diacare.28.12.2890

[39] Schafer S, Zernecke A. CD8(+) T Cells in Atherosclerosis. Cells 2020;10:37. 33383733 10.3390/cells10010037 PMC7823404

