# Supplementary material for: Associations of the inflammasome-dependent cytokine IL-18 with risk of coronary heart disease
Source: Cardiovasc Res. 2026 Jun 23;122(11):1506–15. doi: 10.1093/cvr/cvag135 (PMC13473969; doi:10.1093/cvr/cvag135)
Supplement: Supplementary file 1 [file cvag135_supplementary_data.docx]

**Supplemental Online Content**

**Associations of the inflammasome-dependent cytokine IL-18 with risk of coronary heart disease**

Harry Björkbacka PhD^1^, Isabel Gonçalves MD^1,2^, Gunnar Engström PhD^1^, Alexandru Schiopu MD^3.4,5^, Anna von Sydow MSc^6^, Maria Ryaboshapkina MSc^6^, Viji Varma PhD^7^, Alastair Moss MD^8^, Angela C. Shore MD^9^, Andrea Natali MD^10^, Faisel Khan MD^11^, Jan Nilsson MD^1^

^1^Department of Clinical Sciences Malmö, Lund University, Sweden, ^2^Department of Cardiology, Skåne University Hospital, Sweden, ^3^ Department of Translational Medicine, Lund University, Malmö, Sweden, ^4^“Nicolae Simionescu” Institute of Cellular Biology and Pathology, Bucharest, Romania, ^5^Department of Internal Medicine, Skåne University Hospital, Lund, Sweden, ^6^AstraZeneca, Mölndal, Sweden, ^7^AstraZeneca, Gaithersburg, USA, ^8^AstraZeneca, Cambridge, UK, ^9^Diabetes and Vascular Medicine, University of Exeter Medical School and NIHR Exeter Clinical Research Facility, Exeter, UK, ^10^Department of Clinical and Experimental Medicine, University of Pisa, Italy, ^11^Division of Cardiovascular Research, University of Dundee, Dundee, UK.

**Table of contents**

**eMethods**

**eResults**

**Table e1.** Baseline characteristics of UK Biobank Plasma Proteomics Project stratified by incident myocardial infarction.

**Table e2.** Baseline characteristics of UK Biobank Plasma Proteomics Project stratified by incident cardiovascular death.

**Table e3.** IL6 and IL18 did not provide added value for risk stratification beyond the clinical risk factors in the UK Biobank.

**Table e4.** Sample size calculation for future studies.

**Figure e1.** STROBE statement.

**Figure e2.** Spearman Rank correlations of IL-18, IL-6 and hsCRP with cardiovascular risk factors in the Malmö Diet and Cancer study.

**Figure e3.** Spearman Rank correlations of IL-18, IL-6 and hsCRP with cardiovascular risk factors in the UK BioBank Plasma Proteomics Project.

**Figure e4.** Kaplan-Meier curve and at-risk table for IL-6 vs incident myocardial infarction in the UK Biobank Plasma Proteomics Project. The plot shows all participants with available OLINK IL-6 measurements at baseline.

**Figure e5.** Kaplan-Meier curve and at-risk table for IL-6 vs incident cardiovascular death in the UK Biobank Plasma Proteomics Project. The plot shows all participants with available OLINK IL-6 measurements at baseline

**Figure e6.** IL-6 was associated with risk of incident myocardial infarction and cardiovascular death in the UK Biobank Plasma Proteomics project.

**Figure e7.** Addition of IL6 or IL18 to clinical risk factors resulted in re-distribution of weights on the clinical risk factors (a), resulting in an alternative but redundant expression of overall risk (b).

**eMethods**

**UK biobank Pharma Proteomics Project**

Plasma proteome measurements were collected in 54,219 participants of the general UK population in the UK biobank using OLINK Explore 3072. Data processing and normalization were conducted as described in the original Sun et al. 2023 publication ^1^. Samples from participants who withdrew their consent, outliers and mislabeled samples were excluded. In addition, all individual protein measurements that did not pass technical assay quality control criteria by OLINK were excluded from downstream analysis resulting in slightly varying number of participants available for individual analyses. Protein measurements were scaled and centered and analyzed in relative quantification units where 1 unit corresponded to 1 standard deviation.

**Covariate and outcome definitions**

Demographics and biochemistry measurements at baseline were obtained from the UK Biobank. The field accession numbers for high sensitivity C-reactive protein and LDL direct were 30710 and 30780, respectively. Non-HDL cholesterol was defined as total cholesterol – direct LDL cholesterol.

Diabetes was defined based on date of diagnosis of type 1 or type 2 diabetes (E10 or E11) or HbA1c >= 6.5% prior to sample collection for proteomics and used as a covariate in Cox regression analysis.

Smoking was defined as current active or occasional smoking based on a combination of entries from medical history and questionnaire at the assessment center at or prior to sample collection for proteomics and used as a covariate in Cox regression analysis.

Myocardial infarction was defined based on ICD-10 codes (any of I21*, I211, I212, I213, I214, I219, I22*, I221, I228, I229, I23*, I231, I232, I233, I234, I235, I236, I238, I241, I252). * denotes any child ICD-10 code here. Prior myocardial infarction was defined as a myocardial infarction within five years before the plasma sample collection date for proteomics and was used as covariate in Cox regression analysis. Myocardial infarction as outcome was defined as the first occurrence of any of the ICD-10 codes listed above after the sample collection date for proteomics.

Cardiovascular death was defined as the first occurrence of any of the following ICD-10 codes: I011, I018, I050, I051, I059, I069, I070, I071, I079, I080, I081, I091, I099, I110, I119, I200, I209, I210, I211, I212, I213, I214, I219, I220, I221, I229, I248, I249, I251, I252, I255, I258, I259, I270, I272, I279, I289, I301, I318, I319, I330, I340, I348, I349, I350, I351, I358, I359, I369, I38*, I420, I421, I426, I429, I441, I442, I443, I453, I458, I459, I460, I469, I471, I472, I48*, I480, I489, I490, I499, I500, I501, I509, I515, I517, I518, I519, Q210, Q213, Q231, Q238, Q248 and R570.

The date participants were considered censored were the date participant left the study, the date of the last follow-up visit or the date of non-cardiac death, whichever occurred first.

**Statistical analysis in the UK Biobank**

Baseline demographics and biochemical characteristics of the participants were presented as number and percentage of patients for binary variables and as median and width of interquartile range for continuous variables. The relationships between IL-6 and IL-18 measurements and the biochemical and demographic characteristics of the participants at baseline were assessed using Spearman rank correlation. The relationships between tertiles of IL-6 and IL-18 and risk of incident myocardial infarction or cardiovascular death were assessed using Cox regression estimated with the Breslow’s method.

Five models were evaluated. Model M1 was univariate and assessed IL-6 or IL-18 as stand-alone risk factors. Models M2 and M3 explored if IL-6 or IL-18 remained a non-redundant risk factor after taking the other inflammation markers into account. Model M2 was adjusted only for hsCRP as a non-specific marker of systemic inflammation. Model M3 was adjusted for IL-6 when evaluating IL-18 and adjusted for IL-18 when evaluating IL-6 as the main risk factor. M4 was adjusted for age and sex. M5 was adjusted for age, sex, non-HDL cholesterol, systolic blood pressure, diabetes, current smoking and prior myocardial infarction. Clinical risk factors (M5) and inflammation-associated risk factors (M2, M3) were evaluated as separate models to avoid collinearity issues within each model and to maximize the number of participants eligible for analysis with available covariate measurements.

The models were estimated using CoxPHFitter functionality in lifelines v 0.29.0 in Python 3.9.5. Cox proportional hazard assumption was checked based on residuals diagnostics. Event rates were calculated for the univariate model and corresponded to the number of incident myocardial infarctions or cardiovascular death per 1,000 person-years of follow-up. Kaplan-Meier survival curves were plotted using the implementation in lifelines v 0.29.0.

**The assessment of added biomarker value**

All Cox regression models for this assessment were fit on the same subset of 43,515 UK Biobank participants with complete set of measurements for all covariates in model M5 (age, sex, non-HDL cholesterol, systolic blood pressure, diabetes, current smoking and prior myocardial infarction) as well as CRP, IL6 and IL18 and information about both myocardial infraction and cardiovascular death outcomes. The first multivariate Cox regression model was fit only on the clinical risk factors (M5) with and without CRP. The second Cox regression model included all covariates from the first model and an added biomarker: IL6, IL18 or both IL6 and IL18, The performance of the second compared to the first model was assessed with time-dependent area under the curve calculated at the end of follow-up (AUC^2^) and net reclassification improvement (NRI^3^). Time dependent AUC was implemented in survivalROC v 1.0.3.1 in R 4.3.1.

**Sample size**

The number of patients required to detect the association between the third tertile of IL6 or IL18 and risk of incident myocardial infarction and death in future studies was calculated based on event frequencies and multivariate hazard ratios given full covariate adjustment for clinical risk factors (M5) and CRP in the general UK Biobank population, power 80% and alpha 0.05 using the Latouche et al. 2004 method^4^ as implemented in the powerSurvEpi version 0.1.5 R package^5^.

**Table e1.** Baseline characteristics of UK Biobank Plasma Proteomics Project stratified by incident myocardial infarction.

|  | **No myocardial infarction (n=49,564)** | **Incident myocardial infarction (n=2,454)** |
| --- | --- | --- |
| Age (years) | 58.00 (13.00) | 63.00 (9.00)*** |
| Males (n (%)) | 22306.0 (45.0) | 1680.0 (68.5)*** |
| Smoking (n (%)) | 5278 (10.6) | 399 (16.3)*** |
| Diabetes (n (%)) | 1628 (3.3) | 281 (11.5)*** |
| BMI (kg/m^2) | 26.71 (5.71) | 28.24 (5.95)*** |
| HbA1c (%) | 3.41 (0.47) | 3.59 (0.55)*** |
| LDL (mmol/L) | 3.50 (1.19) | 3.17 (1.37)*** |
| HDL (mmol/L) | 1.40 (0.51) | 1.21 (0.43)*** |
| TG (mmol/L) | 1.47 (1.08) | 1.76 (1.23)*** |
| Systolic BP (mm Hg) | 136.00 (25.00) | 141.00 (24.50)*** |
| Diastolic BP (mm Hg) | 81.50 (13.50) | 82.00 (15.00) |
| hsCRP (mg/L) | 1.33 (2.11) | 1.83 (2.94)*** |
| IL-6 (NPX) | -0.02 (1.34) | 0.44 (1.29)*** |
| IL-18 (NPX) | -0.01 (1.35) | 0.28 (1.26)*** |
| Prior MI within 5 years to baseline (n(%)) | 205 (0.4) | 179 (7.3)*** |

Continuous variables are presented as median (width of the interquartile range). ***p<0.001 Mann-Whitney U test for continuous variables and Chi-squared test for binary variables.

**Table e2.** Baseline characteristics of UK Biobank Plasma Proteomics Project stratified by incident cardiovascular death.

|  | **No cardiovascular death (n=49,373)** | **Cardiovascular death**  **(n=1,537)** |
| --- | --- | --- |
| Age (years) | 58.00 (13.00) | 65.00 (7.00)*** |
| Males (n (%)) | 22340.0 (45.2) | 1106.0 (72.0)*** |
| Smoking (n (%)) | 5260 (10.7) | 297 (19.3)*** |
| Diabetes (n (%)) | 1591 (3.2) | 291 (18.9)*** |
| BMI (kg/m^2) | 26.73 (5.70) | 28.58 (7.06)*** |
| HbA1c (%) | 3.41 (0.47) | 3.69 (0.78)*** |
| LDL (mmol/L) | 3.50 (1.19) | 2.97 (1.27)*** |
| HDL (mmol/L) | 1.40 (0.51) | 1.21 (0.45)*** |
| TG (mmol/L) | 1.47 (1.09) | 1.67 (1.18)*** |
| Systolic BP (mm Hg) | 136.00 (25.00) | 141.50 (26.00)*** |
| Diastolic BP (mm Hg) | 81.50 (13.50) | 79.75 (15.00)*** |
| hsCRP (mg/L) | 1.33 (2.10) | 2.27 (4.10)*** |
| IL-6 (NPX) | -0.02 (1.34) | 0.74 (1.26)*** |
| IL-18 (NPX) | -0.01 (1.35) | 0.40 (1.37)*** |
| Prior MI within 5 years to baseline (n(%)) | 263 (0.5) | 73 (4.7)*** |

Continuous variables are presented as median (width of the interquartile range). ***p<0.001 Mann-Whitney U test for continuous variables and Chi-squared test for binary variables.

**Table e3.** IL6 and IL18 did not provide added value for risk stratification beyond the clinical risk factors in the UK Biobank. Clinical risk factors were age, sex, non-HDL cholesterol, systolic blood pressure, diabetes, current smoking and prior myocardial infarction. NRI stands for net reclassification performance. zero or near-zero improvement in AUC and NRI indicates no added biomarker value. Model comparison was performed on a smaller subset of 43,515 participants with complete covariate and outcome data compared to the main analysis.

|  |  | **Cardiovascular death** | | |
| --- | --- | --- | --- | --- |
|  | **Metric** | **IL6** | **IL18** | **IL6 & IL18** |
| Without CRP | Clinical risk factors AUC | 0.81 | 0.81 | 0.81 |
|  | Biomarker + clinical risk factors AUC | 0.82 | 0.81 | 0.82 |
|  | AUC Improvement | 0.01 | 0.00 | 0.02 |
|  | NRI Event | 0.03 | 0.01 | 0.03 |
|  | NRI Censored | 0.00 | 0.00 | 0.00 |
|  | NRI Total | 0.03 | 0.01 | 0.04 |
| With CRP | Clinical risk factors and CRP AUC | 0.82 | 0.82 | 0.82 |
|  | Biomarker + clinical risk factors and CRP AUC | 0.82 | 0.82 | 0.82 |
|  | AUC Improvement | 0.01 | 0.00 | 0.01 |
|  | NRI Event | 0.01 | 0.00 | 0.02 |
|  | NRI Censored | 0.00 | 0.00 | 0.00 |
|  | NRI Total | 0.01 | 0.00 | 0.02 |
|  |  | **Myocardial infarction** | | |
|  | **Metric** | **IL6** | **IL18** | **IL6 & IL18** |
| Without CRP | Clinical risk factors AUC | 0.74 | 0.74 | 0.74 |
|  | Biomarker + clinical risk factors AUC | 0.75 | 0.74 | 0.75 |
|  | AUC Improvement | 0.01 | 0.00 | 0.01 |
|  | NRI Event | 0.01 | 0.00 | 0.02 |
|  | NRI Censored | 0.00 | 0.00 | 0.00 |
|  | NRI Total | 0.01 | 0.00 | 0.02 |
| With CRP | Clinical risk factors and CRP AUC | 0.74 | 0.74 | 0.74 |
|  | Biomarker + clinical risk factors and CRP AUC | 0.75 | 0.74 | 0.75 |
|  | AUC Improvement | 0.01 | 0.00 | 0.01 |
|  | NRI Event | 0.01 | 0.00 | 0.01 |
|  | NRI Censored | 0.00 | 0.00 | 0.00 |
|  | NRI Total | 0.01 | 0.00 | 0.01 |

**Table e4.** Sample size calculation for future studies. Multivariate hazard ratios were calculated given full covariate adjustment for age, sex, non-HDL cholesterol, systolic blood pressure, diabetes, current smoking and prior myocardial infarction and C-reactive protein in the UK Biobank and assumed to represent the true effect size. T3 and T1 stand for the third and first tertile, respectively. R2 is the proportion of variability in IL-6 or IL-18 that is explained by the prognostic index calculated from the clinical covariates and CRP.

| **IL-6** | **MI** | **CV death** |
| --- | --- | --- |
| Power | 0.80 | 0.80 |
| Alpha | 0.05 | 0.05 |
| Proportion of patients in T3 | 1/3 | 1/3 |
| Multivariate HR T3 vs T1 | 1.64 | 2.41 |
| R2 | 0.10 | 0.10 |
| If proportion of events as in UK BB | 0.05 | 0.03 |
| N | 3,368 | 1,702 |
| *If proportion of events as in MCD* | 0.13 | 0.136 |
| *N* | 376 | 1,238 |
| **IL-18** | **MI** | **CV death** |
| Power | 0.80 | 0.80 |
| Alpha | 0.05 | 0.05 |
| Proportion of patients in T3 | 1/3 | 1/3 |
| Multivariate HR T3 vs T1 | 1.19 | 1.30 |
| R2 | 0.05 | 0.05 |
| If proportion of events as in UK BB | 0.05 | 0.03 |
| N | 25,649 | 17,581 |
| *If proportion of events as in MCD* | 0.13 | 0.136 |
| *N* | 9,428 | 3,883 |

**Figure e1.** STROBE statement

|  | | **Item No** | **Recommendation** | **Page No** |
| --- | --- | --- | --- | --- |
| **Title and abstract** | | 1 | (*a*) Indicate the study’s design with a commonly used term in the title or the abstract | 3 |
|  |  |  | (*b*) Provide in the abstract an informative and balanced summary of what was done and what was found | 3 |
| **Introduction** | | | | |
| Background/rationale | | 2 | Explain the scientific background and rationale for the investigation being reported | 5,6 |
| Objectives | | 3 | State specific objectives, including any prespecified hypotheses | 5,6 |
| **Methods** | | | | |
| Study design | | 4 | Present key elements of study design early in the paper | 6 |
| Setting | | 5 | Describe the setting, locations, and relevant dates, including periods of recruitment, exposure, follow-up, and data collection | 6-9, e3 |
| Participants | | 6 | (*a*) Give the eligibility criteria, and the sources and methods of selection of participants. Describe methods of follow-up | 6,7, e3 |
|  |  |  | (*b*) For matched studies, give matching criteria and number of exposed and unexposed |  |
| Variables | | 7 | Clearly define all outcomes, exposures, predictors, potential confounders, and effect modifiers. Give diagnostic criteria, if applicable | 6,7, e3 |
| Data sources/ measurement | | 8* | For each variable of interest, give sources of data and details of methods of assessment (measurement). Describe comparability of assessment methods if there is more than one group | 6,7, e3 |
| Bias | | 9 | Describe any efforts to address potential sources of bias | 15 |
| Study size | | 10 | Explain how the study size was arrived at | 9,10,15  e3-5 |
| Quantitative variables | | 11 | Explain how quantitative variables were handled in the analyses. If applicable, describe which groupings were chosen and why | 9,10, e4 |
| Statistical methods | | 12 | (*a*) Describe all statistical methods, including those used to control for confounding | 9, e4 |
|  |  |  | (*b*) Describe any methods used to examine subgroups and interactions | e4 |
|  |  |  | (*c*) Explain how missing data were addressed | 7 |
|  |  |  | (*d*) If applicable, explain how loss to follow-up was addressed | NA |
|  |  |  | (*e*) Describe any sensitivity analyses | NA |
| **Results** | | | |  |
| Participants | | 13* | (a) Report numbers of individuals at each stage of study—eg numbers potentially eligible, examined for eligibility, confirmed eligible, included in the study, completing follow-up, and analysed | 25,27 |
|  |  |  | (b) Give reasons for non-participation at each stage | NA |
|  |  |  | (c) Consider use of a flow diagram | NA |
| Descriptive data | | 14* | (a) Give characteristics of study participants (eg demographic, clinical, social) and information on exposures and potential confounders | 24, e6 |
|  |  |  | (b) Indicate number of participants with missing data for each variable of interest | NA |
|  |  |  | (c) Summarise follow-up time (eg, average and total amount) | 10,11 |
| Outcome data | | 15* | Report numbers of outcome events or summary measures over time | 25,27 |
| Main results | 16 | (*a*) Give unadjusted estimates and, if applicable, confounder-adjusted estimates and their precision (eg, 95% confidence interval). Make clear which confounders were adjusted for and why they were included | | 26,28 |
|  |  | (*b*) Report category boundaries when continuous variables were categorized | | 25,27 |
|  |  | (*c*) If relevant, consider translating estimates of relative risk into absolute risk for a meaningful time period | | NA |
| Other analyses | 17 | Report other analyses done—eg analyses of subgroups and interactions, and sensitivity analyses | | NA |
| **Discussion** | | | | |
| Key results | 18 | Summarise key results with reference to study objectives | | 14,15 |
| Limitations | 19 | Discuss limitations of the study, taking into account sources of potential bias or imprecision. Discuss both direction and magnitude of any potential bias | | 15,17 |
| Interpretation | 20 | Give a cautious overall interpretation of results considering objectives, limitations, multiplicity of analyses, results from similar studies, and other relevant evidence | | 14-17 |
| Generalisability | 21 | Discuss the generalisability (external validity) of the study results | | 16 |
| **Other information** | | | | |
| Funding | 22 | Give the source of funding and the role of the funders for the present study and, if applicable, for the original study on which the present article is based | | 18 |

**Figure e2.** Spearman Rank correlations of IL-18, IL-6 and hsCRP with cardiovascular risk factors in the Malmö Diet and Cancer study.

N 4,661. All correlations are p<0.05. Correlations |r|>0.06 are p<0.001. Reference ranges for correlation coefficients: r |0-0.3| - weak, r |0.31-0.6| - moderate, >0.6 strong.

**Figure e3.** Spearman Rank correlations of IL-18, IL-6 and hsCRP with cardiovascular risk factors in the UK BioBank Plasma Proteomics Project.

N>50,000. Correlations |r|>0.014 are p<0.001. Reference ranges for correlation coefficients: r |0-0.3| - weak, r |0.31-0.6| - moderate, >0.6 strong.

**Figure e4.** Kaplan-Meier curve and at-risk table for IL-6 vs incident myocardial infarction in the UK Biobank Plasma Proteomics Project. The plot shows all participants with available OLINK IL-6 measurements at baseline. Lowest tertile <-0.430 NPX, mid tertile ≥-0.430–0.432 NPX, highest tertile >0.432 NPX.

**Figure e5.** Kaplan-Meier curve and at-risk table for IL-6 vs incident cardiovascular death in the UK Biobank Plasma Proteomics Project. The plot shows all participants with available OLINK IL-6 measurements at baseline. Lowest tertile <-0.430 NPX, mid tertile ≥-0.430–0.432 NPX, highest tertile >0.432 NPX.

**Figure e6.** IL-6 was associated with risk of incident myocardial infarction and cardiovascular death in the UK Biobank Plasma Proteomics project.

Cox proportional hazard regression models (M) were adjusted as follows: M1 was unadjusted; M2 was adjusted for hsCRP; M3 was adjusted for IL-18; M4 was adjusted for age, sex; M5 was adjusted for age, sex, non-HDL cholesterol, systolic blood pressure, diabetes, current smoking and prior myocardial infarction. *p<0.05, **p<0.01 and ***p<0.001. T1-lowest tertile, T2 – middle tertile, T3-highest tertile. CV- cardiovascular. Models M1, M2, M4 and M5 were estimated on the subset of participants who had all M5 covariate measurements available at baseline. M3 was estimated on a larger set of all participants with available OLINK IL-6 and IL-18 measurements at baseline.

**Figure e7.** Addition of IL6 or IL18 to clinical risk factors resulted in re-distribution of weights on the clinical risk factors (**a**), resulting in an alternative but redundant expression of overall risk (**b**). Representative example for in multivariate Cox regression for IL18 vs risk of cardiovascular death the UK Biobank. Prognostic index is the weighted sum of all risk factors for each participant, where weight is non-exponentiated regression coefficient. One data point on the plot is one participant. Model comparison was performed on a smaller subset of 43,515 participants with complete covariate and outcome data compared to the main analysis.

| a. **covariate** | **multivariate HR** | **multivariate HR** |
| --- | --- | --- |
| Male vs female | 2.42 | 2.32 |
| Per 1 year of age | 1.12 | 1.12 |
| With vs without prior MI | 2.74 | 2.74 |
| With vs without diabetes | 3.30 | 3.20 |
| Smoking vs non-smoking | 2.23 | 2.20 |
| Per 1 mm/Hg systolic blood pressure | 1.00 | 1.00 |
| Per 1 mmol/L non-HDL cholesterol | 0.80 | 0.79 |
| CRP >= 2 vs <2 mg/L | 1.99 | 1.92 |
|  | + IL18 T2 vs T1 | 1,09 |
|  | + IL18 T3 vs T1 | 1,30 |

References

1.	Sun BB, Chiou J, Traylor M, Benner C, Hsu YH, Richardson TG, Surendran P, Mahajan A, Robins C, Vasquez-Grinnell SG, Hou L, Kvikstad EM, Burren OS, Davitte J, Ferber KL, Gillies CE, Hedman AK, Hu S, Lin T, Mikkilineni R, Pendergrass RK, Pickering C, Prins B, Baird D, Chen CY, Ward LD, Deaton AM, Welsh S, Willis CM, Lehner N, Arnold M, Worheide MA, Suhre K, Kastenmuller G, Sethi A, Cule M, Raj A, Alnylam Human Genetics, AstraZeneca Genomics Initiative, Biogen Biobank Team, Bristol Myers Squibb, Genentech Human Genetics, GlaxoSmithKline Genomic Sciences, Pfizer Integrative Biology, Population Analytics of Janssen Data Sciences, Regeneron Genetics Center, Burkitt-Gray L, Melamud E, Black MH, Fauman EB, Howson JMM, Kang HM, McCarthy MI, Nioi P, Petrovski S, Scott RA, Smith EN, Szalma S, Waterworth DM, Mitnaul LJ, Szustakowski JD, Gibson BW, Miller MR, Whelan CD. Plasma proteomic associations with genetics and health in the UK Biobank. *Nature* 2023;**622**:329-338.

2.	Heagerty PJ, Lumley T, Pepe MS. Time-dependent ROC curves for censored survival data and a diagnostic marker. *Biometrics* 2000;**56**:337-344.

3.	Pencina MJ, D'Agostino RB, Sr., D'Agostino RB, Jr., Vasan RS. Evaluating the added predictive ability of a new marker: from area under the ROC curve to reclassification and beyond. *Stat Med* 2008;**27**:157-172; discussion 207-112.

4.	Latouche A, Porcher R, Chevret S. Sample size formula for proportional hazards modelling of competing risks. *Stat Med* 2004;**23**:3263-3274.

5.	Qiu W, Chavarro J, Lazarus R, Rosner B, Ma J. powerSurvEpi: Power and Sample Size Calculation for Survival Analysis of Epidemiological Studies. R package version 0.1.5, 2025.
